# Course of disease in multifocal choroiditis lacking sufficient immunosuppression: a case report

**DOI:** 10.1186/s13256-016-1069-2

**Published:** 2016-10-24

**Authors:** Katharina Schroeder, Tobias Meyer-ter-Vehn, Heidi Fassnacht-Riederle, Rainer Guthoff

**Affiliations:** 1 Department of Ophthalmology, University of Duesseldorf, Moorenstr. 5, 40225 Duesseldorf, Germany; 2 Department of Ophthalmology, University of Wuerzburg, Wuerzburg, Germany; 3 Department of Ophthalmology, Stadtspital Triemli, Zürich, Switzerland

**Keywords:** Multifocal choroiditis, Chorioretinal lesions, Secondary CNV, Bevacizumab, Systemic immunosuppression, Case report

## Abstract

**Background:**

Multifocal choroiditis with panuveitis is a rare disease. The educational merit of this case presentation results from the good documentation and the impressive ocular fundus pictures.

**Case presentation:**

We illustrate the 3-year course of disease in a 22-year-old myopic white woman with multifocal choroiditis with panuveitis and secondary choroidal neovascularization. The activity of the disease was evaluated clinically by optical coherence tomography and fluorescein angiography. Choroidal neovascularization was treated by intravitreal bevacizumab (2.5 mg/0.1 ml). Our patient lacked systemic therapy for the first 11 months because of noncompliance.

**Conclusions:**

The case is remarkable as the delayed onset of peripheral lesions and the additional existence of high myopia made diagnosis difficult. In addition, it demonstrates that full outbreak of disease with multiple central and peripheral fundus lesions and secondary choroidal neovascularization can develop without systemic treatment.

## Background

Multifocal choroiditis (MFC) with panuveitis is a rare, recurrent white dot syndrome affecting myopic women in their third to fourth decades. Symptoms include blurred vision, photopsia, or scotoma [1]. Clinical findings comprise vitritis and multiple, small, round, yellowish lesions at the level of the retinal pigment epithelium and choriocapillaris at the posterior pole and in the periphery. The presence of anterior uveitis or vitritis seems to have prognostic implications [2]. Characteristically the lesions are hypofluorescent in fluorescein angiography (FA) and indocyanine green angiography (ICGA) [1]. During the course of the disease the lesions become hyperpigmented [3]. Treatment encompasses systemic or periocular steroids and immunosuppression. Secondary choroidal neovascularization (CNV) occurs in 27 to 46 % of cases [1, 3–6]. It can be treated by intravitreal anti-vascular endothelial growth factor (VEGF) application, laser photocoagulation, photodynamic therapy, or surgical excision [3, 4].

We present a 3-year course of a young myopic white woman with MFC and secondary CNV. The activity of the disease was evaluated clinically by optical coherence tomography (OCT; Stratus, Carl-Zeiss-Meditec, Inc.) and FA. Intravitreal bevacizumab (2.5 mg/0.1 ml) was injected after informed consent was given. Retreatment depended on visual acuity (VA), OCT, and FA findings. The educational merit of this case presentation results from the good documentation and the impressive ocular fundus pictures. In addition, it displays why diagnosis can be difficult in the beginning and emphasizes the therapeutic importance of systemic immunosuppression.

## Case presentation

**Fig. 1:**
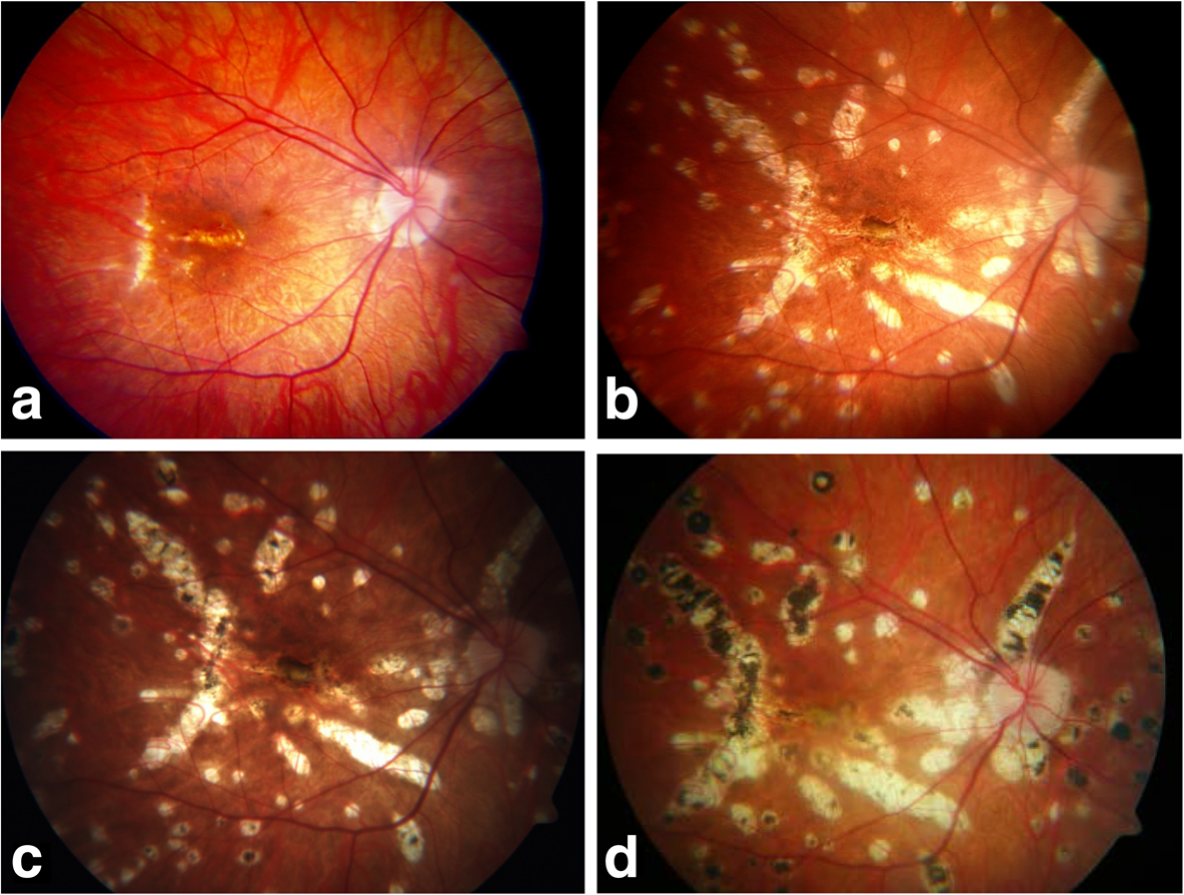
Representative fundus images of right eye. **a** Initially apart from lacquer cracks and a discrete hemorrhage no peripheral lesions are visible. **b** Eleven months later, multiple yellow to gray lesions at the level of the retinal pigment epithelium occur at the posterior pole and midperipherally. **c** On immunosuppression no additional lesions occur 14 months and (**d**) 37 months later. Instead, lesions become increasingly hyperpigmented indicating cicatrization

**Fig. 2:**
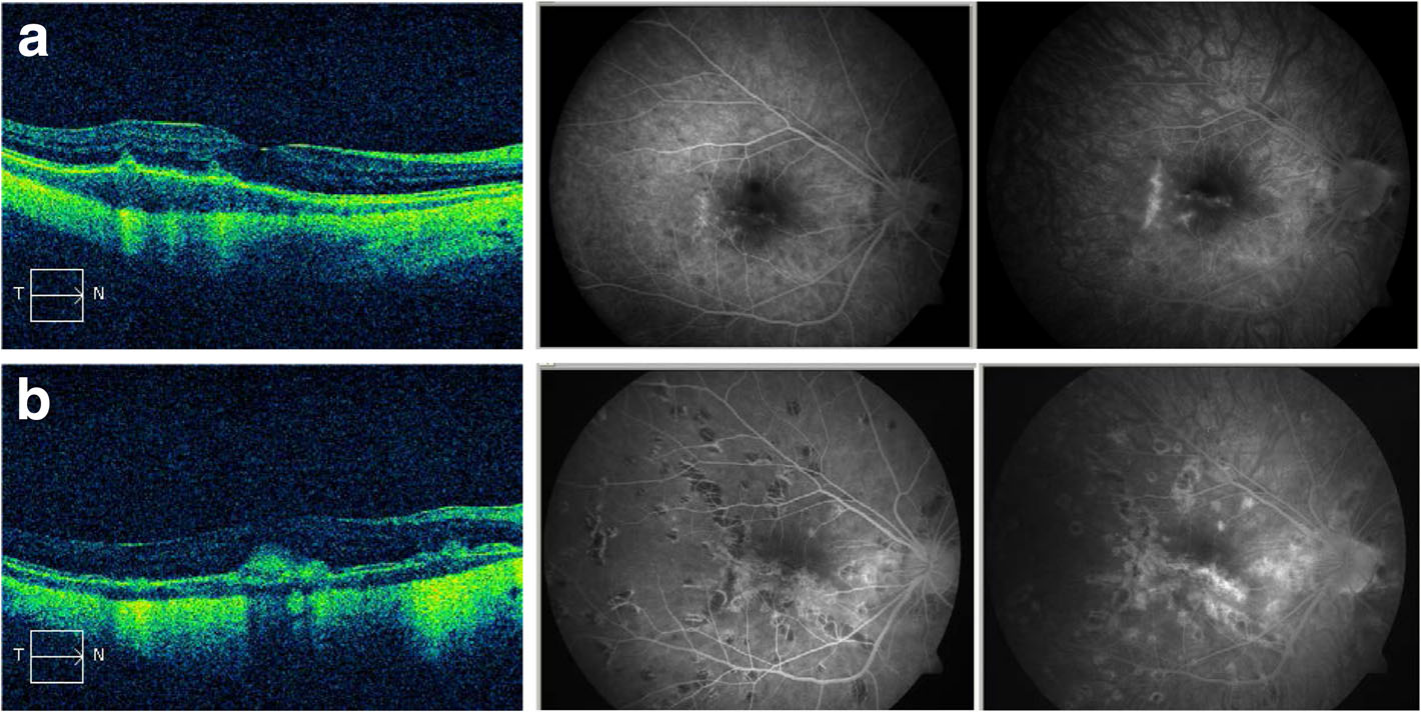
Optical coherence tomography and fluorescein angiography images of the right eye (**a**) first presentation: retinal pigment epithelium detachment on optical coherence tomography and discrete subretinal fluid can be detected. On fluorescein angiography discrete hyperfluorescence with a discrete late leakage in terms of a choroidal neovascularization near to the lacquer crack is visible. Furthermore, fluorescein angiography reveals discrete roundish hypofluorescent lesions, which are clinically unapparent (Fig. 1a) but correspond to future lesions. **b** Eleven months later: hypofluorescent lesions have considerably increased in number and were hyperfluorescent in late phase fluorescein angiography. A discrete macular late leakage is found in the right eye consistent with a reactivated choroidal neovascularization

A 22-year-old white woman presented with decreased VA and a central floater in her right eye (OD) for 2 weeks. Apart from bilateral high myopia of –14 diopter ophthalmological, her general and family history were unremarkable. Her Snellen VA was 20/32 in her OD and 20/20 in her left eye (OS). Her intraocular pressure was normotonic. Funduscopy revealed a myopic fundus with lacquer crack and small macular hemorrhage in her OD (Fig. 1a). OCT showed discrete subretinal fluid suspicious for CNV. FA was consistent with CNV (Fig. 2a). She did not show up for further examinations for personal reasons.

She presented again 11 months later with a loss of VA and floaters in her OD. In the meantime she had received intravitreal bevacizumab (2.5 mg/0.1 ml) for CNV in her OS elsewhere. Her VA was 20/200 in her OD and 20/32 in her OS. A clinical examination showed vitreous cells and roundish yellow to gray chorioretinal lesions in the central and midperipheral fundus bilaterally. A small subretinal hemorrhage (Fig. 1b) was found in her OD and macular fibrosis was found in her OS. In FA the multiple lesions were hypofluorescent in early phase and hyperfluorescent in late phase. A discrete macular leakage corresponded to a subfoveal CNV in her OD (Fig. 2b). Borreliosis, toxoplasmosis, and syphilis were ruled out serologically. A clinical examination did not show any evidence of tuberculosis or sarcoidosis and MFC with panuveitis was diagnosed. A combined treatment of oral steroids (prednisolone 60 mg daily administered orally, tapered off gradually) and intravitreal bevacizumab in her OD was started. Subsequently, cyclosporine was administered orally. One month later VA in her OS dropped to 20/100 with a corresponding late leakage in FA which was compatible with CNV reactivation. Bevacizumab was re-injected in her OS.

During the following 15 months the number of inflammatory lesions remained constant with ongoing pigmentation (Fig. 1c) indicating absent active inflammation. Her VA increased to 20/100 in her OD and 20/25 in her OS. Systemic immunosuppression was discontinued. However, 1 month and 7 months later CNV recurred in her OS. Bevacizumab was re-injected twice. Three years after her first presentation her VA was 20/40 in her OD and 20/200 in her OS.

## Conclusions

The case reported is of educational merit because of its impressive ocular fundus pictures. In addition, it is remarkable in terms of diagnostics because of the delayed onset of peripheral lesions and the additional existence of high myopia that made diagnosis of MFC difficult. CNV in MFC occurs in 27 to 46 % of cases [1, 3–6] and can be the first symptom [3]. MFC lesions may be clinically occult, which impedes clinical diagnosis in early stages of the disease. At our patient’s presentation FA revealed only discrete multifocal peripheral lesions, which were clearly evident 11 months later. ICGA could have been an additional diagnostic tool displaying hypofluorescent lesions that were not visible clinically or on FA. The hypofluorescence is considered to represent non-perfusion areas of the choriocapillaris [1]. Because of the myopic fundus with lacquer cracks it was difficult to diagnose CNV funduscopically. Late leakage in FA was discrete. Spaide *et al*. stated that in some cases differentiation between active inflammatory lesions and CNV may be impossible even with multimodal imaging as both can cause infiltrative lesions with breakdown of the blood-barrier [7]. Our patient’s response to intravitreal bevacizumab confirmed the existence of CNV.

Treatment options for inflammation in MFC are oral, periocular, or intraocular steroids along with immunosuppressive agents [6]. Our patient lacked systemic therapy for the first 11 months because further examinations were rejected. Therefore this case demonstrates that full outbreak of disease with multiple lesions of the central and peripheral fundus can develop without systemic treatment. Notably these lesions remained asymptomatic. On treatment with prednisolone administered orally and consecutive cyclosporine administered orally, her inflammation subsided illustrating the need of systemic immunosuppression. The number of lesions stagnated and a progressive pigmentation indicating cicatrization occurred.

Our case shows that CNV reactivation may occur despite effective immunosuppression, at least at the beginning of therapy. Nevertheless, immunosuppression is supposed to prevent CNV by reducing the inflammatory stimulus for neo-angiogenesis [5, 8]. Before starting and after cessation of systemic immunosuppression, CNV recurred at frequent intervals bilaterally. This emphasizes the importance of long-term effective immunosuppression and short-term control in MFC.

In our case secondary CNV responded to intravitreal bevacizumab. This is in accordance with studies where intravitreal anti-VEGF was beneficial for CNV secondary to MFC in terms of effectivity and safety [3, 5, 6, 9, 10]. A small number of re-injections – two at OD and three at OS over 3 years – were sufficient for CNV control. This is consistent with the literature [3]. Other CNV treatment approaches such as argon laser photocoagulation, photodynamic therapy, and surgical excision are regarded to be inferior [1, 3, 4, 10]. In accordance with the literature, VA in our patient improved after intravitreal bevacizumab applications and no adverse events occurred [3].

In conclusion, MFC is a rare recurrent disease that predominantly affects young myopic women. Therefore a careful dilated fundus examination including the periphery is mandatory in all myopic women with CNV. As clinical diagnosis can be difficult, FA and ICGA are recommended for suspect cases. CNV occurrence seems to be associated with insufficient immunosuppression. When CNV is present it can be treated effectively with anti-VEGF therapy requiring only few re-injections.

## Abbreviations

CNV: Choroidal neovascularization
FA: Fluorescein angiography
ICGA: Indocyanine green angiography
MFC: Multifocal choroiditis
OCT: Optical coherence tomography
OD: Right eye
OS: Left eye
VA: Visual acuity
VEGF: Vascular endothelial growth factor
